# Valorization of Distilled Paint Waste as a Modifier of Polymer Matrices Based on ABS, PP and PS

**DOI:** 10.3390/ma19194167

**Published:** 2026-09-29

**Authors:** Bartłomiej Jagodziński, Krzysztof Moraczewski, Tomasz Karasiewicz, Krzysztof Szabliński

**Affiliations:** Faculty of Materials Engineering, Kazimierz Wielki University in Bydgoszcz, Chodkiewicza 30 Str., 85-064 Bydgoszcz, Poland; tomakara@ukw.edu.pl (T.K.); k.sza@ukw.edu.pl (K.S.)

**Keywords:** paint waste, polymer composites, mechanical properties, thermal analysis, circular economy

## Abstract

This study evaluates the feasibility of incorporating a specific batch of distilled paint waste into acry-lonitrile–butadiene–styrene (ABS), polypropylene (PP), and polystyrene (PS) matrices at nominal feed contents of 10, 20, and 30 wt.%. After drying and grinding, the waste was compounded by extrusion and specimens were produced by injection molding. Melt-flow behavior, density, impact and tensile properties, thermal stability, thermal transitions, and dynamic-mechanical response were investigated. The effect of waste addition was strongly matrix dependent. In PS, MFR increased from 9.52 to 24.85 g/10 min between neat PS and the 30 wt.% nominal formulation, while Tg decreased from 106.4 to 81.6 °C. TGA showed non-monotonic, matrix-dependent changes in thermal stability. Mechanical performance generally deteriorated with increasing nominal waste content; for ABS, tensile strength decreased from 42.21 to 26.17 MPa and impact strength from 17.51 to 0.23 kJ/m^2^. DMA confirmed matrix-dependent changes in stiffness and relaxation behavior. The results show that the investigated waste batch can be processed with all three ther-moplastic matrices, but increasing waste addition involves clear mechanical and thermal trade-offs.

## 1. Introduction

Contemporary materials engineering is increasingly oriented toward solutions consistent with the principles of the circular economy, in which post-production waste is regarded not only as an environmental problem but also as a potential source of secondary raw materials. Paint waste generated during coating application, installation washing, paint booth cleaning, and solvent recovery constitutes a particularly important group. Such waste streams usually have a complex and heterogeneous composition, including resins, pigments, mineral fillers, auxiliary additives, solvent residues, and products of partial or complete curing of coating systems [1,2]. This chemical and morphological complexity makes paint waste a difficult material from the perspective of both classification and further management, while at the same time making it an interesting research object in terms of material recovery [1,2].

From the perspective of the circular economy, the key issue is not only recycling understood as material recovery, but also maintaining the highest possible material value by reintroducing waste streams into manufacturing processes. This approach is consistent with the concept of reducing the consumption of virgin raw materials, minimizing emissions, and closing material loops. In relation to polymer materials, it requires the design of systems that enable stable processing, controlled quality, and predictable performance properties [3,4,5,6,7,8,9,10].

The importance of this issue results not only from technological considerations but also from the legal framework in force in the European Union. Directive 2008/98/EC is of fundamental importance in this respect, as it establishes the waste hierarchy and defines the basic concepts related to recovery and recycling [11]. This approach is complemented by the European Commission guidelines on waste classification, which emphasize the need to assess the actual composition and properties of waste when assigning it to appropriate categories, including hazardous waste [12]. In relation to coating processes, Directive 2010/75/EU on industrial emissions is also important, as it covers industrial activities involving the use of organic solvents, including coating processes [13]. In turn, Regulation (EU) 2024/1157 regulates the rules for waste shipments, which is important for the organization and control of waste streams directed to recovery or disposal [14].

In the case of polymer materials, the transition from a linear model to a circular model is particularly demanding, because plastic and composite waste is characterized by diverse composition, processing history, the presence of technological additives, and variable quality of the secondary raw material. For this reason, current research directions include both mechanical and chemical recycling, as well as the development of composites in which waste fractions function as fillers, modifiers, or functional components [15,16,17,18,19,20,21,22].

In this context, distilled paint waste, i.e., the residue remaining after the recovery of volatile fractions, mainly solvents, is a particularly interesting research material. Compared with untreated paint waste, such a material contains fewer volatile components, but a relatively higher proportion of the solid fraction composed of resins, pigments, fillers, and formulation additives. From the perspective of polymer processing technology, this means that, after appropriate drying and grinding, such waste may be considered as a potential filler or modifier of the properties of a thermoplastic matrix. The study by Viguri et al. demonstrated that the characterization and distillation of alkyd paint waste make it possible to assess the technical feasibility of solvent recovery and to rationally plan further handling of the process residue [23].

The literature shows that paint waste has so far been analyzed mainly from the perspective of its generation, characterization, and general management methods. In their review of paint sludge from the automotive industry, Salihoglu and Salihoglu discussed its sources, physicochemical properties, and the most commonly considered further treatment routes, such as drying, incineration, co-processing, or use in other materials [1]. The authors emphasized that the key problem is the high variability in the composition of such waste, which makes it difficult to implement a single universal management pathway. Ruffino et al. presented a more recent review concerning methods for the pretreatment of paint sludge, particularly dewatering, drying, and further possibilities for material or energy recovery [2]. Their analysis clearly indicates that waste preparation prior to further use largely determines the actual technological applicability of such material.

A more direct reference to the use of paint waste in new materials can be found in the work of Nakouzi et al. [24]. The authors proposed converting paint sludge into ceramic composites, which could subsequently be used as reinforcing materials in metal and polymer systems. Although these studies did not concern classical thermoplastic composites, they were conceptually important because they demonstrated that components of paint sludge can be recovered and used as a functional material phase. A similar direction of valorization was presented by Abhyankar, Edmonds, and Easteal [25], who investigated the possibility of using waste powder coatings as binders in composite materials. Their results showed that waste coating material may act not only as an inert additive, but also actively participate in the formation of a new composite system.

The studies closest to the topic of thermoplastic composites containing waste coating phases are those by Kismet and co-workers [26,27]. In their 2017 paper, the authors analyzed the possibility of using thermoset powder coating recyclates as fillers in LLDPE. These wastes were first chemically modified, and then their processability with the polymer matrix and their influence on the mechanical properties of the composites were evaluated [26]. In a subsequent study published in 2021, the same researchers used three different thermoset electrostatic powder coating wastes as fillers for LDPE at contents of 10, 20, and 30 wt.% [27]. The obtained materials were evaluated in terms of mechanical, thermal, and morphological properties. The authors showed that, with increasing waste content, tensile strength decreased, whereas flexural strength increased and some impact properties improved, which confirmed the possibility of using this type of waste as a functional polymer filler [27].

Similar conclusions are also supported by more recent studies on HDPE systems containing waste powder coating. Ariantika, Chalid, and Pangesty [28] investigated HDPE-based composites containing waste coating powder and the coupling agent PP-g-MA. The study analyzed the morphology, mechanical properties, and thermal behavior of the materials, demonstrating that appropriate selection of the waste content and compatibilizer may improve interactions between the matrix and the waste phase [28]. A different but also important perspective was presented by Yeğin and Öksüz [29], who analyzed the recycling of painted and coated ABS in PC/ABS blends with ABS-g-MA. The authors examined the effect of the compatibilizer on mechanical properties and fracture morphology, showing that plastics previously covered with paint layers can be effectively reintroduced into the material cycle [29].

The cited publications confirm that paint and coating wastes may be considered as potential components of new materials; however, their influence on composite properties depends on the type of waste, the degree of its processing, the proportion of the waste phase, and the chemical nature of the polymer matrix [1,2,26,27,28,29]. Nevertheless, there are still relatively few studies concerning the direct use of distilled paint waste as an additive for common thermoplastics with different chemical structures, such as ABS, PP, and PS. For this reason, research on such systems is of both cognitive and practical importance, as it makes it possible to assess whether waste after solvent recovery can function as a useful material component rather than solely as a problematic by-product [1,2,11].

This is also confirmed by studies concerning other waste streams introduced into polymer matrices. Among other things, the possibility of using waste brewer’s yeast biomass as a biofiller for polylactide and waste photopolymer printing matrices as a filler for polypropylene has been demonstrated. The results of these studies indicate that effective waste management in composites requires assessment not only of processing feasibility, but also of the influence of the waste phase on interfacial adhesion, porosity, thermal stability, and mechanical properties [30,31].

From the perspective of designing polymer composites containing waste phases, particle dispersion, filler wettability by the polymer melt, interfacial adhesion, and the possibility of void and agglomerate formation are of particular importance. These factors determine the efficiency of stress transfer, the course of crystallization, viscoelastic behavior, and the thermal stability of the material. Therefore, the interpretation of results for systems containing distilled paint waste should be placed not only in the literature concerning coating wastes, but also in the broader context of polymer composites with waste fillers and biofillers [32,33,34,35,36,37,38,39,40].

## 2. Materials and Methods

Polypropylene (PP) Moplen EP548U (LyondellBasell, Rotterdam, The Netherlands) was selected as one of the matrices for the new polymer composites. According to the technical data sheet, this polymer is characterized by a density of 0.90 g/cm^3^, a melt flow rate of 70 g/10 min (230 °C/2.16 kg), tensile strength of 28 MPa, elongation at break of 30%, notched impact strength of 4 kJ/m^2^, and Young’s modulus of 1450 MPa. Polystyrene (PS) PS-GP154 (Synthos, Oświęcim, Poland) was characterized by a density of 1.04 g/cm^3^, a melt flow rate (MFR) of 9–10 g/10 min (200 °C/5 kg), and unnotched impact strength of 10 kJ/m^2^.

Acrylonitrile-butadiene-styrene terpolymer (ABS) Terluran GP-35 (INEOS Styrolution, Antwerpen, Belgium) was used as the third polymer matrix. According to the material data sheet, this polymer is characterized by a density of 1.04 g/cm^3^, a melt flow rate of 3.1 g/10 min (200 °C/5 kg), and Charpy impact strength of 130 kJ/m^2^.

**Figure 1:**
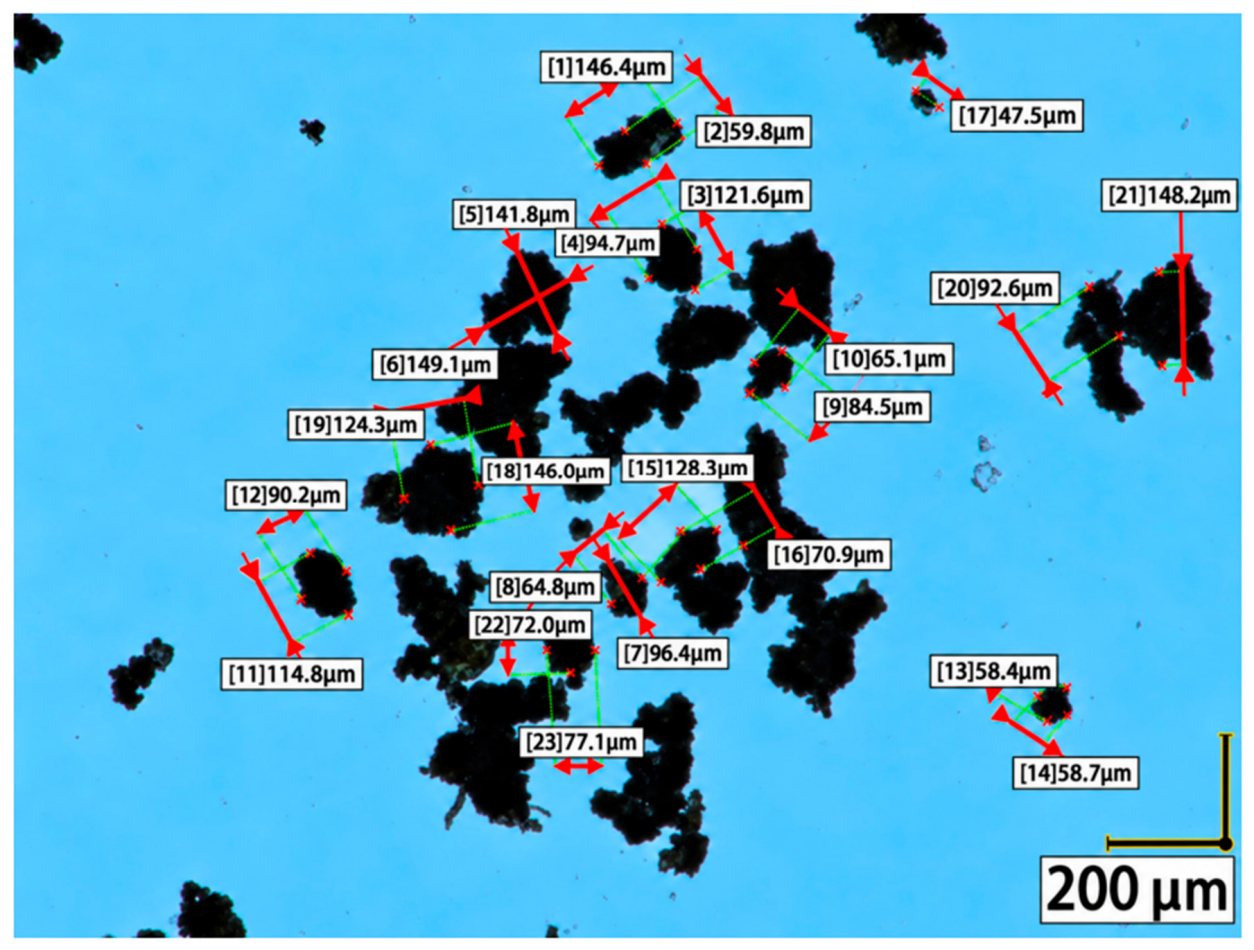
Microscopic appearance of distilled paint waste after drying and grinding.

The PP, PS, and ABS matrices were filled with distilled paint waste obtained from MIPA Polska Sp. z o.o. (Solec Kujawski, Poland).The distilled paint waste used in this study originated from an actual industrial waste stream comprising different material and product batches, including residues from decorative, automotive, and industrial coatings, aerosol paints, and wood coatings. Consequently, the material was multicomponent and heterogeneous in nature, which is characteristic of real-world paint waste subjected to a distillation process. The waste was in the form of paint sludge, which was left at room temperature for approximately two months to allow the remaining volatile components to evaporate. Subsequently, the material was dried in a laboratory oven for one week at 50 °C and ground in a laboratory mill designed for polymer materials. ‘The microscopic appearance of the ground filler is shown in Figure 1.

Microscopic image analysis of the ground distilled paint waste revealed particles with irregular shapes and a broad size distribution. Based on measurements of 23 representative particles, the mean characteristic particle size was determined to be 98.0 ± 34.1 µm, with values ranging from 47.5 to 149.1 µm. The median particle size was 92.6 µm. The observed morphology and the relatively wide particle-size range indicate the geometrical heterogeneity of the powder, which is characteristic of material obtained by mechanical grinding of solid paint waste.

The investigated polymer materials were produced using two processing methods: extrusion and injection molding. Composite granulates were prepared by extrusion. Ground distilled paint waste was added at contents of 10, 20, and 30 wt.% to polymer matrices (ABS, PP, PS) during the extrusion process. The extrusion process was carried out using a W25-30D single-screw extruder (Metalchem, Toruń, Poland) equipped with an intensive-mixing screw featuring additional kneading elements. The screw rotation speed was maintained at a constant 200 rpm. The temperature profile across the four barrel zones was set to 165, 185, 195, and 195 °C, respectively, while the die temperature was maintained at 195 °C. This same extrusion temperature profile was applied to the neat ABS, PP, and PS matrices and to all corresponding composites containing 10, 20, and 30 wt.% of distilled paint waste, as summarized in Table 1. The extrudates were cooled by an air stream and subsequently pelletized using a knife granulator. Ground distilled paint waste was added at nominal feed contents of 10, 20, and 30 wt.% during extrusion, calculated from the initial masses of polymer and dried waste introduced into the process. The retained waste fraction after melt processing was not quantified; therefore, all wt.% values and sample designations used in this study refer to nominal feed compositions rather than verified final compositions.

Three composite formulations were prepared for each polymer, containing 10, 20, and 30 wt.% of ground distilled paint waste. The designations of the samples used are presented in Table 2. The properties of the new composites were compared with those of the neat polymer materials (samples A, B, C).

Test specimens were then produced from the obtained granulate by injection molding. Prior to injection, the granulate was dried in a laboratory dryer at 50 °C for 24 h. Injection molding was carried out using a TRX 80 Eco screw injection molding machine (Tederic, Taizhou, China) equipped with a mold for standardized dumbbell-shaped specimens. The specimen dimensions were as follows: thickness 4.0 ± 0.2 mm, gauge section width 10.0 ± 0.2 mm, gauge length 80.0 ± 0.2 mm, and total length above 150 mm. Injection temperatures are presented in Table 3. The mold was thermostated at 35 °C. The injection and holding pressures were 60 bar and 58 bar, respectively. The cooling time of the molded part in the mold was 60 s.

The densities of the materials were measured using an MVP-D160E gas pycnometer (Quantachrome Instruments, Boynton Beach, FL, USA), with helium as the measuring gas. The density value was calculated as the arithmetic mean of three measurements of the same sample.

Melt flow rate (MFR) measurements were carried out using an MP600 capillary plastometer (Tinius Olsen, Horsham, PA, USA). The flow rate measurements were performed at 230 °C under a load of 2.16 kg for PP-based compositions, at 200 °C for PS, and at 235 °C for ABS, under a load of 5 kg. For each material, 12 consecutive extrudate cuts were collected; the MFR was calculated from 10 values after discarding the highest and lowest results.

Impact strength tests (Charpy method) were carried out using an XJ 5Z impact tester (Liangong, Jinan, China) with a 2 J hammer at a drop velocity of 2.9 m/s. Unnotchedspecimens measuring 80 mm × 10 mm × 4 mm, cut from the tensile test specimens, weretested. The unnotched impact strength (au) was determined, with twelve repetitions permaterial. The final reported value was the arithmetic mean.

Tensile strength tests were carried out using an Instron 3367 universal testing machine (Instron, Norwood, MA, USA) at a crosshead speed of 20 mm/min, using standardized dumbbell-shaped specimens. Strain was determined from the crosshead displacement; no extensometer was used during the measurements. The elastic modulus (E) was calculated automatically by the Instron testing software Bluehill 2 based on the recorded force–crosshead displacement data. No separate strain interval for modulus determination was manually defined by the operator. Therefore, the reported modulus values should be regarded as instrument-derived estimates rather than extensometer-based modulus values determined strictly according to the ISO 527 procedure. Eight repetitions were performed for each analyzed sample series, and the final values of the measured parameters were calculated as the arithmetic mean. The tensile tests were otherwise conducted in accordance with PN-EN ISO 527-1 [41] and PN-EN ISO 527-2 [42].

Thermomechanical analysis (DMA) was performed using a Q800 dynamic mechanical analyzer (TA Instruments, New Castle, WA, USA). Measurements were conducted in three-point bending mode over a temperature range of 30–150 °C with a heating rate of 3 °C/min. Bar-shaped specimens (60 × 10 × 4 mm), cut from the measuring section of the paddle-shaped samples obtained via injection molding, were used. The specimens were supported at two points and subjected to an oscillatory load applied at the midpoint of the span. The applied displacement amplitude was 15 μm, and the oscillation frequency was 1 Hz.

Differential scanning calorimetry (DSC) analysis was performed using a Q200 calorimeter (TA Instruments, New Castle, WA, USA) under a nitrogen atmosphere. The measurements were carried out on unmodified polymers and composites containing 10, 20, and 30 wt.% distilled paint waste, namely A, A10, A20, and A30 for the ABS matrix; B, B10, B20, and B30 for the PP matrix; and C, C10, C20, and C30 for the PS matrix. The sample mass ranged from 3.1 to 3.6 mg.

The measurements were conducted over a temperature range from −50 to 210 °C at heating and cooling rates of 10 °C/min. Each sample was subjected to three consecutive stages of the temperature program: first heating, cooling, and second heating. The first heating cycle was applied to minimize the influence of the thermal history introduced during prior material processing and was not used for the quantitative comparison of the thermal properties of the individual compositions. The thermal parameters were analyzed based on the cooling and second-heating curves. The obtained DSC curves were used to determine the glass transition temperature (Tg), crystallization temperature (Tc), crystallization enthalpy (ΔHc), melting temperature (Tm), melting enthalpy (Δ*H_m_*), and, for the PP-based composites, the degree of crystallinity (*X_c_*).

(1) $$X_{c}=\left(\frac{∆H_{m}}{∆H_{m100\%}\cdot (1-x)}\right)\cdot 100\%$$

where Δ*H_m_* is the melting enthalpy of the sample, Δ*H_m_*_100%_ is the melting enthalpy of 100% crystalline PP, assumed to be 207 J/g, and *x* is the nominal mass fraction of waste. The degree of crystallinity should be determined and interpreted only for systems with a PP matrix. Because the retained waste fraction after melt processing was not quantified, the nominal waste feed fraction was used as an approximation in the crystallinity calculation; therefore, the reported *X_c_* values should be regarded as approximate.

Thermogravimetric analysis (TGA) was performed using a Q500 thermogravimeter (TA Instruments, New Castle, WA, USA) under a nitrogen atmosphere in the temperature range of 25–1000 °C, at a heating rate of 10 °C/min. The sample mass ranged from 17.9 to 23.2 mg. Based on the recorded TG and DTG curves, the onset decomposition temperature (Tonset), the temperature of maximum mass loss rate (Tmax), the mass loss (Δm), and the final residue at 1000 °C were determined for each distinct degradation stage. Additionally, the temperature corresponding to a 50% loss of the initial sample mass (T50%) was determined.

## 3. Results and Discussion

The incorporation of distilled paint waste into the ABS and PP matrices resulted in a systematic increase in density with increasing waste content. For ABS, the density increased from 1.04 to 1.10 g/cm^3^, whereas for PP it increased from 0.90 to 0.98 g/cm^3^ (Table 4).

**Figure 2:**
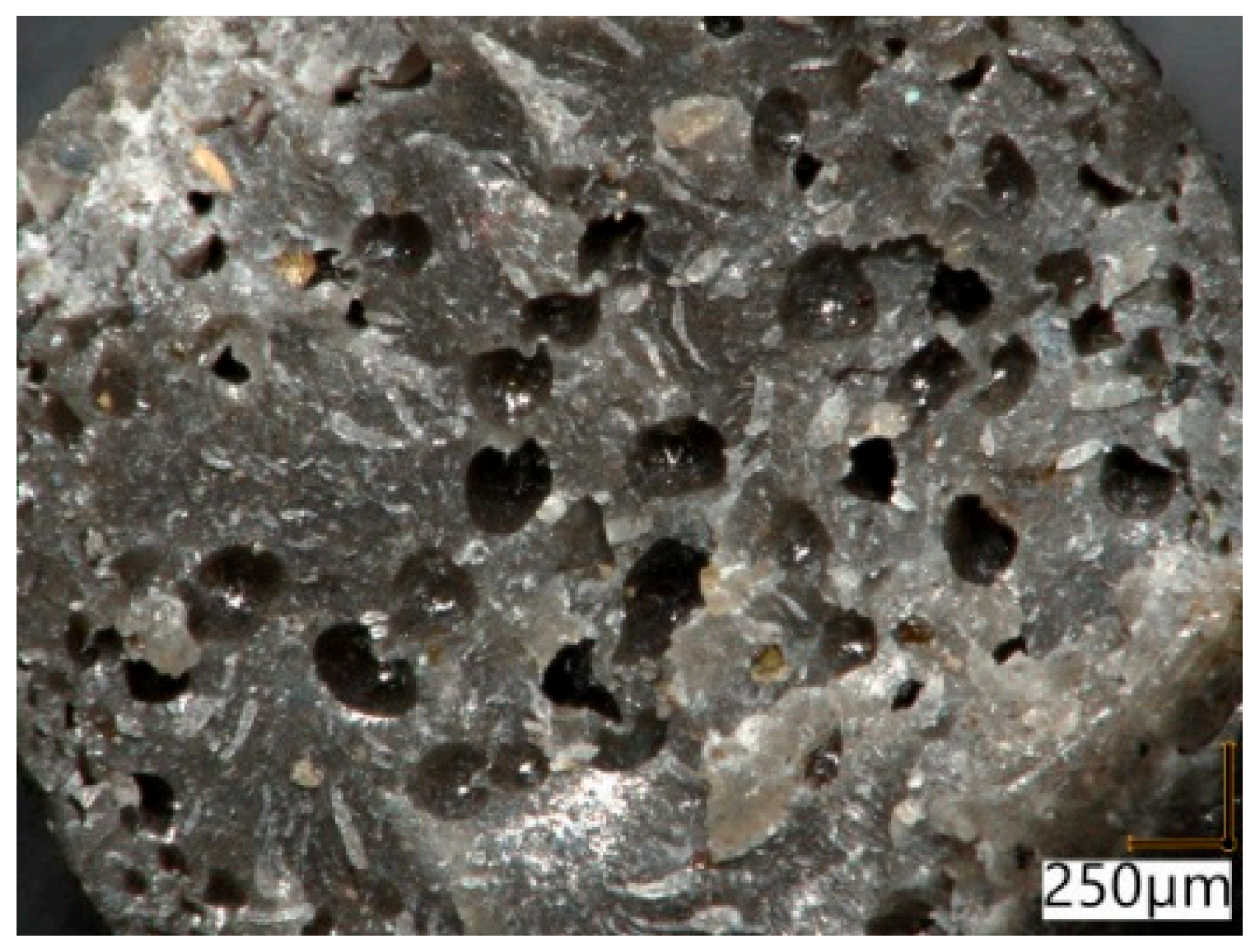
Microphotograph of the granulate structure of PS samples containing distilled paint waste.

A different trend was observed for PS. The skeletal density determined by helium pycnometry was 1.05 g/cm^3^ for C, 0.90 g/cm^3^ for C10, 0.94 g/cm^3^ for C20, and 0.96 g/cm^3^ for C30. Because helium pycnometry determines skeletal density and helium can penetrate accessible pore space, these values cannot be directly interpreted as evidence of porosity or increased free volume. The optical micrograph in Figure 2 qualitatively shows voids in the granulate; however, their contribution to bulk porosity was not quantified in this study.

For ABS, MFR increased from 18.38 g/10 min for the neat polymer to 23.97 and 24.53 g/10 min for A10 and A20, respectively, and decreased to 20.30 g/10 min for A30 (Table 5). These results demonstrate a non-monotonic change in melt-flow behavior with waste content. Because MFR is a single-point flow index, the observed changes cannot by themselves distinguish among rheological, compositional, degradation-related, or feeding effects.

MFR exhibited a non-monotonic dependence on waste content in the B series. It decreased from 64.77 g/10 min for B to 39.06 g/10 min for B10, then increased to 46.45 and 58.30 g/10 min for B20 and B30, respectively, remaining below the value measured for B. These results indicate a nonlinear change in melt-flow behaviour under the applied test conditions. However, elucidating the mechanism underlying these changes is beyond the scope of MFR measurements and requires further rheological investigations and detailed characterisation of the chemical composition of the waste.

In the C series, MFR increased monotonically with increasing waste content, from 9.52 g/10 min for C to 17.28, 22.40, and 24.85 g/10 min for C10, C20, and C30, respectively. This indicates progressively easier melt flow under the applied test conditions; however, MFR alone cannot uniquely identify the origin of this effect or confirm a plasticising effect of the waste constituents. The observed porosity is an additional factor that may affect the uniformity of material feeding during measurement, although its influence on the measured MFR values was not quantified.

**Figure 3:**
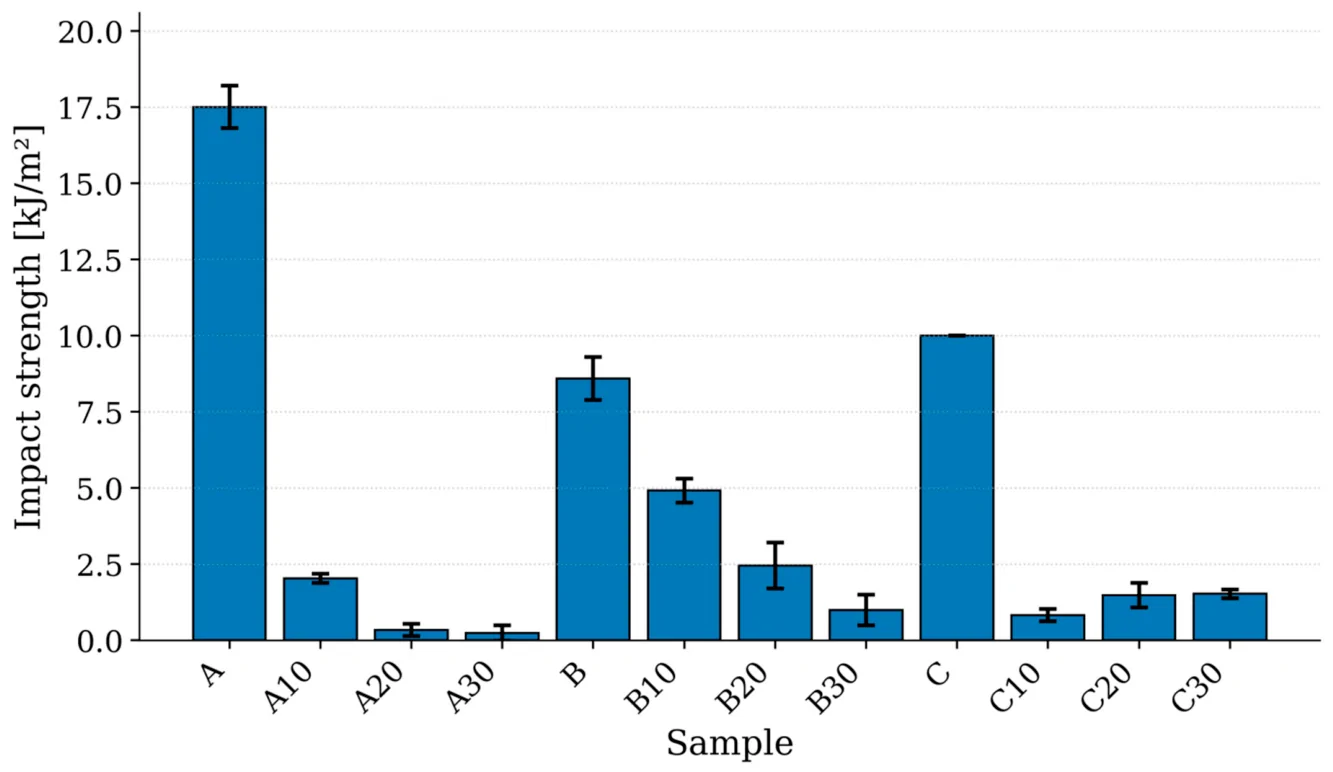
Impact strength of the tested materials.

Analysis of Charpy impact strength results showed that the effect of the additive depends on the type of polymer matrix (Figure 3).

The most pronounced monotonic deterioration in impact properties was observed for the ABS-based materials. The mean impact strength decreased from 17.51 kJ/m^2^ for sample A to 2.03, 0.33, and 0.23 kJ/m^2^ for A10, A20, and A30, respectively. Thus, the incorporation of only 10 wt.% of the additive reduced the impact strength by approximately 88%, while at 30 wt.% the reduction reached approximately 99%. These results indicate a substantial loss of the ability of the ABS-based materials to absorb energy under dynamic loading. For the PP-based materials, the reduction was more gradual but remained monotonic. The mean impact strength decreased from 8.59 kJ/m^2^ for sample B to 4.91, 2.45, and 0.99 kJ/m^2^ for B10, B20, and B30, respectively. These values correspond to reductions of approximately 43, 71, and 88% relative to the unmodified PP matrix. PP therefore exhibited greater tolerance to the initial addition of the waste than ABS, although the progressive decrease in impact strength indicates increasing brittleness with increasing additive content. A different, non-monotonic trend was observed for the PS-based materials. Sample C exhibited a mean impact strength of 9.97 kJ/m^2^, whereas the value decreased sharply to 0.82 kJ/m^2^ for C10, corresponding to a reduction of approximately 92%. The mean impact strength subsequently increased to 1.48 kJ/m^2^ for C20 and 1.52 kJ/m^2^ for C30. However, even the highest value recorded among the PS composites remained approximately 85% lower than that of neat PS. Moreover, the difference between C20 and C30 was small, suggesting stabilization of the impact response rather than a continued improvement with increasing waste content. Therefore, the higher impact strength of C20 and C30 compared with C10 should not be interpreted as an improvement relative to the unmodified PS matrix. Without statistical confirmation, the differences among the modified PS samples should be interpreted cautiously. Overall, the addition of distilled paint waste markedly reduced the impact strength of all three polymer matrices. ABS exhibited the strongest monotonic deterioration over the investigated composition range, PP showed a progressive but less abrupt reduction, and PS displayed a pronounced initial decrease followed by a slight recovery at higher additive contents. Nevertheless, all modified PS samples remained substantially more brittle than neat PS. The results therefore indicate that the incorporation of the additive generally promotes brittle behavior and limits the resistance of the investigated materials to dynamic loading [33].

Analysis of the static tensile test results showed that the incorporation of distilled paint waste into thermoplastic matrices adversely affects the mechanical properties of the investigated materials (Table 6).

For ABS, the maximum tensile stress decreased from 42.21 MPa for sample A to 32.27 MPa, 28.48 MPa, and 26.17 MPa for A10, A20, and A30, respectively. These changes corresponded to reductions of approximately 23.5%, 32.5%, and 38.0% relative to the unmodified matrix. A reduction in tensile strength was therefore observed even at the lowest waste content investigated and became more pronounced as the waste content increased. Concurrently, the strain at maximum stress decreased from 3.46% for A to 2.53%, 2.24%, and 2.15% for the corresponding composites. An even greater relative change was observed in strain at break, which decreased from 7.00% to 2.84%, 2.28%, and 2.16%, respectively. These results indicate a reduced capacity of the specimens to undergo deformation before fracture, particularly after the maximum stress had been reached. This interpretation is supported by the convergence of σB towards σM and εB towards εM with increasing waste content. For A, the stress at break was 33.47 MPa, distinctly below the maximum stress. By contrast, these values were nearly identical for A30, at 26.16 MPa and 26.17 MPa, respectively. Together with the small difference between εB and εM, this indicates that fracture occurred almost immediately after the maximum stress was reached. Meanwhile, the automatically determined modulus remained at a similar level: 1557 MPa for A and 1566 MPa, 1548 MPa, and 1550 MPa for A10, A20, and A30, respectively. Thus, no systematic decrease in this parameter was observed under the procedure used. The deterioration in the properties of ABS primarily involved tensile strength and strain at break, rather than a pronounced reduction in the automatically determined modulus.

In the PP series, the maximum tensile stress decreased from 24.81 MPa for B to 20.19 MPa for B10, 19.02 MPa for B20, and 16.55 MPa for B30. The corresponding reductions relative to the unmodified matrix were approximately 18.6%, 23.3%, and 33.3%. These changes were accompanied by a decrease in the automatically determined modulus from 1071 MPa to 779 MPa, 708 MPa, and 661 MPa, respectively. Unlike ABS, the PP series therefore exhibited concurrent reductions in tensile strength and modulus. The strain at maximum stress increased from 6.56% for B to 7.98%, 8.78%, and 8.60% for B10, B20, and B30, respectively. However, this did not indicate an increase in the overall capacity for deformation before fracture. The strain at break, εB, decreased from 23.16% for the matrix to 8.49%, 10.08%, and 9.90% for the composites. Waste incorporation primarily restricted the extent of further deformation after the maximum stress had been reached. The changes in εB among the composites were non-monotonic: the highest mean value was obtained for B20, despite its lower tensile strength compared with B10. Increasing waste content should therefore not be described as causing a uniform deterioration in all mechanical properties of PP. The distinction between σM and σB is particularly important for this series. The stress at break of B10 was 19.93 MPa, slightly higher than the value of 19.12 MPa obtained for B. At the same time, the maximum stress of B10 was lower than that of the matrix. A higher σB therefore does not indicate improved tensile strength; instead, it reflects a different material response between the attainment of maximum stress and fracture.

In the series of PS-based composites, increasing paint waste content was accompanied by a decrease in the measured parameter values. The strain at maximum stress decreased with increasing waste content, whereas the strain at break remained similar for C, C10, and C20. A higher mean strain at break was observed for C30, together with greater scatter in the results. This increase occurred alongside a reduction in tensile strength and does not provide sufficient evidence of improved fracture resistance. Among the investigated composites, changes in the automatically determined modulus were relatively small. The results therefore primarily indicate a reduced stress-bearing capacity and a change in deformation behaviour prior to fracture, without conclusively establishing the mechanisms responsible for these effects.

**Figure 4:**
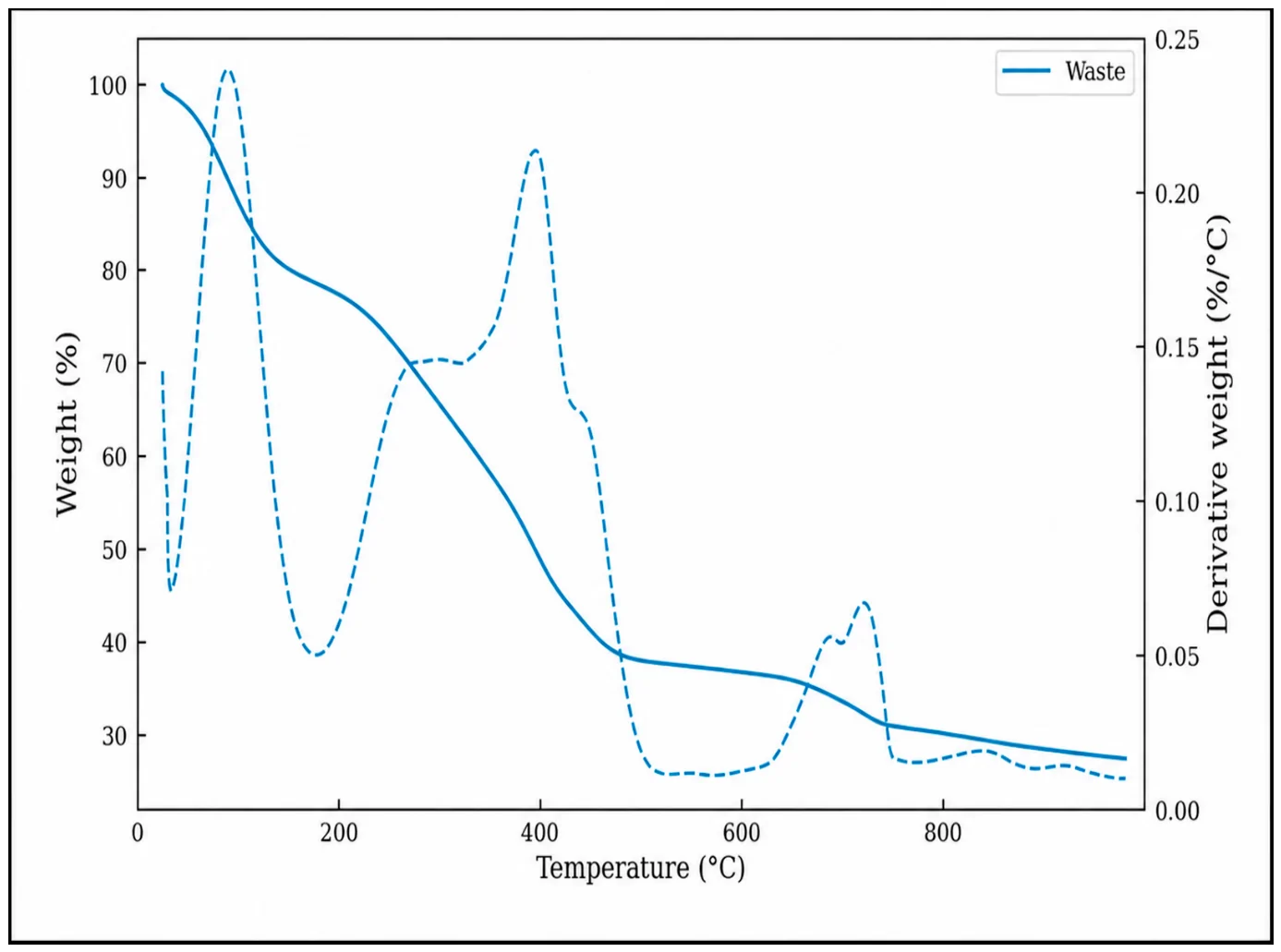
TG/DTG curves of distilled paint waste. Solid lines represent the TG curves, whereas dashed lines represent the DTG curves.

Thermogravimetric analysis of the distilled paint waste revealed a clearly multistage mass-loss behavior (Figure 4). Based on the TG and DTG curves, three main degradation regions were distinguished, with onset temperatures of approximately 25, 177, and 572 °C, respectively.

The first mass-loss stage occurred in the temperature range of 25–177 °C, with Tonset = 25 °C and the maximum mass-loss rate observed at Tmax = 89.4 °C. This stage corresponded to a mass loss of 21.38%. The result indicates the presence of a substantial volatile fraction released at relatively low temperatures. However, without direct identification of the evolved species, this mass loss cannot be unequivocally attributed to residual solvents or other specific low-molecular-weight compounds. From a processing perspective, this stage is particularly important because it occurs below the maximum extrusion temperature used in this study, i.e., 195 °C. This indicates that part of the volatile fraction may be released during melt processing, potentially affecting process stability and the structure of the resulting composites.

The second, quantitatively dominant mass-loss stage occurred in the range of approximately 177–572 °C, with Tonset = 177 °C and Tmax = 395 °C. The mass loss in this region was approximately 41.5%, making it the main degradation stage of the investigated waste. Owing to the heterogeneous origin of the material and the lack of evolved-gas analysis, this stage should be interpreted conservatively as the main mass-loss region, without assigning it to specific resins, oligomers, binders, or other organic constituents.

The third mass-loss stage was observed in the range of approximately 572–981 °C, with Tonset = 572 °C and a DTG maximum at Tmax = 721 °C. This stage was considerably less intense and corresponded to a mass loss of approximately 9% of the initial sample mass. It indicates further transformations of thermally more resistant constituents remaining after the preceding degradation stages. At the end of the measurement, approximately 27.5% of the initial sample mass remained.

Overall, the TG/DTG results indicate that the distilled paint waste undergoes thermal transformations over a broad temperature range and contains a considerable fraction released at relatively low temperatures. Of particular importance is the 21.38% mass loss below 177 °C, as this occurs within a temperature range close to that used during composite processing. Therefore, the presence of this fraction should be taken into account when interpreting the processing behavior and initial thermal stability of the waste-containing materials.

Under an inert atmosphere, the thermal degradation behavior of the individual polymer matrices depends on their chemical structure. In the case of ABS, the degradation process is more complex owing to the multicomponent structure of the copolymer, which comprises a styrene–acrylonitrile phase and polybutadiene segments. PP degradation is associated primarily with thermal scission of the main polymer chain and radical reactions leading to the formation of lower-molecular-weight products. In the case of PS, chain scission and depolymerization processes predominate, resulting in the formation of volatile degradation products. Therefore, the effect of distilled paint waste addition on the TG/DTG behavior should be analyzed individually for each polymer matrix used.

**Figure 5:**
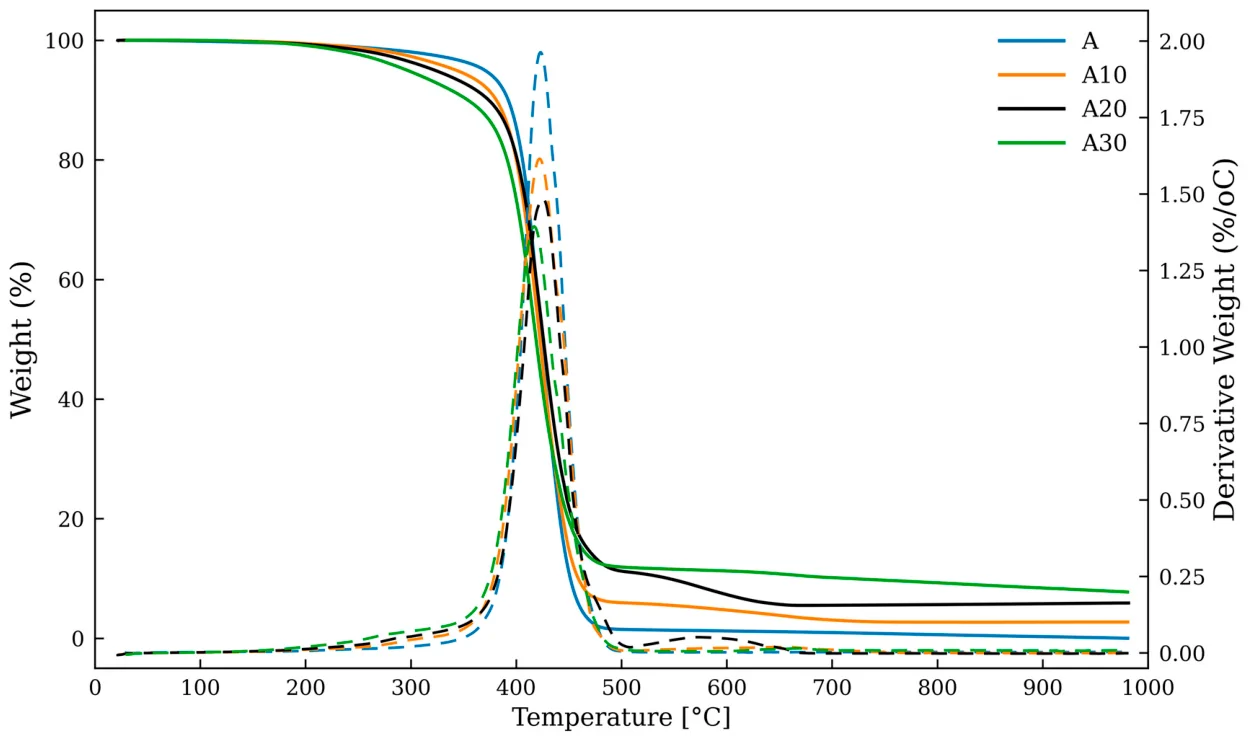
TG/DTG curves of ABS-based samples (A, A10, A20, A30). Solid lines represent the TG curves, whereas dashed lines represent the DTG curves.

Thermogravimetric analysis showed that neat ABS (A) undergoes essentially single-stage thermal degradation and was characterized by Tonset = 398.6 °C, Tmax = 423.0 °C, and T50% = 423.9 °C. The incorporation of distilled paint waste primarily shifted the onset of the main mass-loss process toward lower temperatures. For samples A10 and A20, the Tonset value was identical and amounted to 391.7 °C, whereas for A30 it decreased to 383.2 °C (Figure 5, Table 7).

The temperature corresponding to the maximum mass-loss rate remained relatively stable for samples containing up to 20% waste: 422.2 °C for A10 and 425.3 °C for A20, compared with 423.0 °C for A. Only at a waste content of 30% was a more pronounced shift inTmax toward a lower temperature observed, reaching 416.9 °C. A similar relationship was observed for T50%, which amounted to 423.9, 422.4, and 425.8 °C for A, A10, and A20, respectively, whereas for A30 it decreased to 418.8 °C.

The results therefore indicate that the waste addition primarily affects the onset of the mass-loss process, while the position of the main ABS degradation stage remains relatively stable up to a waste content of 20%. The largest changes in the thermal parameters were observed for sample A30. At the same time, the total mass loss decreased from 99.97% for A to 97.26, 94.08, and 92.24% for A10, A20, and A30, respectively. This trend is consistent with the increasing contribution of waste-derived constituents remaining after the main degradation stage; however, based solely on measurements performed under a nitrogen atmosphere, these residues should not be directly identified as an inorganic fraction.

For sample B, Tonset = 416.3 °C, Tmax = 449.3 °C, and T50% = 438.1 °C were determined. In contrast to the ABS system, the effect of waste addition on the PP degradation parameters was non-monotonic. Tonset increased to 432.2 °C for B10, then decreased to 410.8 °C for B20, while for B30 it amounted to 427.0 °C.

**Figure 6:**
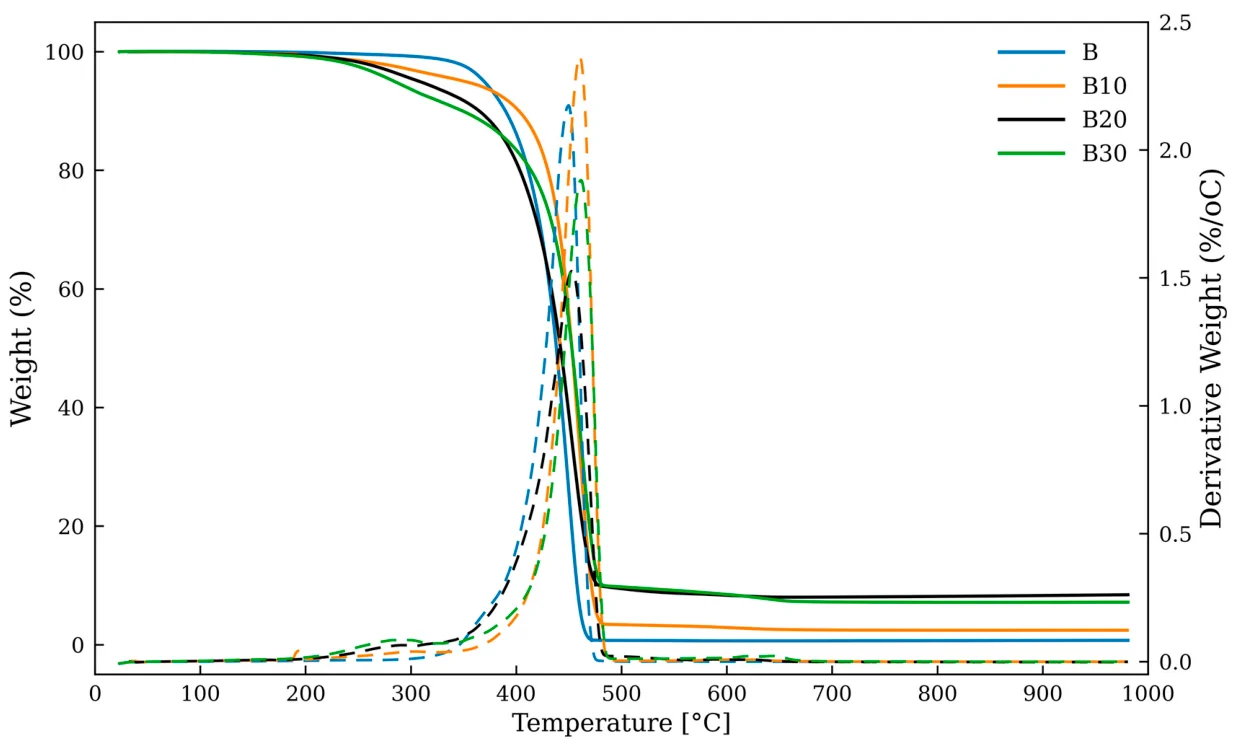
TG/DTG curves of PP-based samples (B, B10, B20, B30). Solid lines represent the TG curves, whereas dashed lines represent the DTG curves.

The Tmax values likewise did not indicate a simple dependence on waste content. The maximum degradation rate occurred at 460.2 °C for B10, 452.9 °C for B20, and 461.4 °C for B30, whereas for neat PP it was 449.3 °C. A similar pattern was observed for T50%: 438.1 °C for B, 452.8 °C for B10, 442.1 °C for B20, and 452.8 °C for B30 (Figure 6).

Based on these results, it is not justified to conclude that increasing the waste content causes a systematic decrease in the thermal stability of PP. The incorporation of waste modifies the degradation behavior; however, the changes in Tonset, Tmax, and T50% are non-monotonic. In particular, samples B10 and B30 exhibited higher Tmax and T50% values than neat PP, whereas B20 showed intermediate values. The total mass loss amounted to 99.34% for B, 97.55% for B10, 91.56% for B20, and 92.84% for B30. These differences confirm the effect of waste content on the mass balance after heating. These differences confirm the effect of waste content on the mass balance after heating. However, the chemical nature of the residue cannot be determined from TGA measurements performed under nitrogen alone.

**Figure 7:**
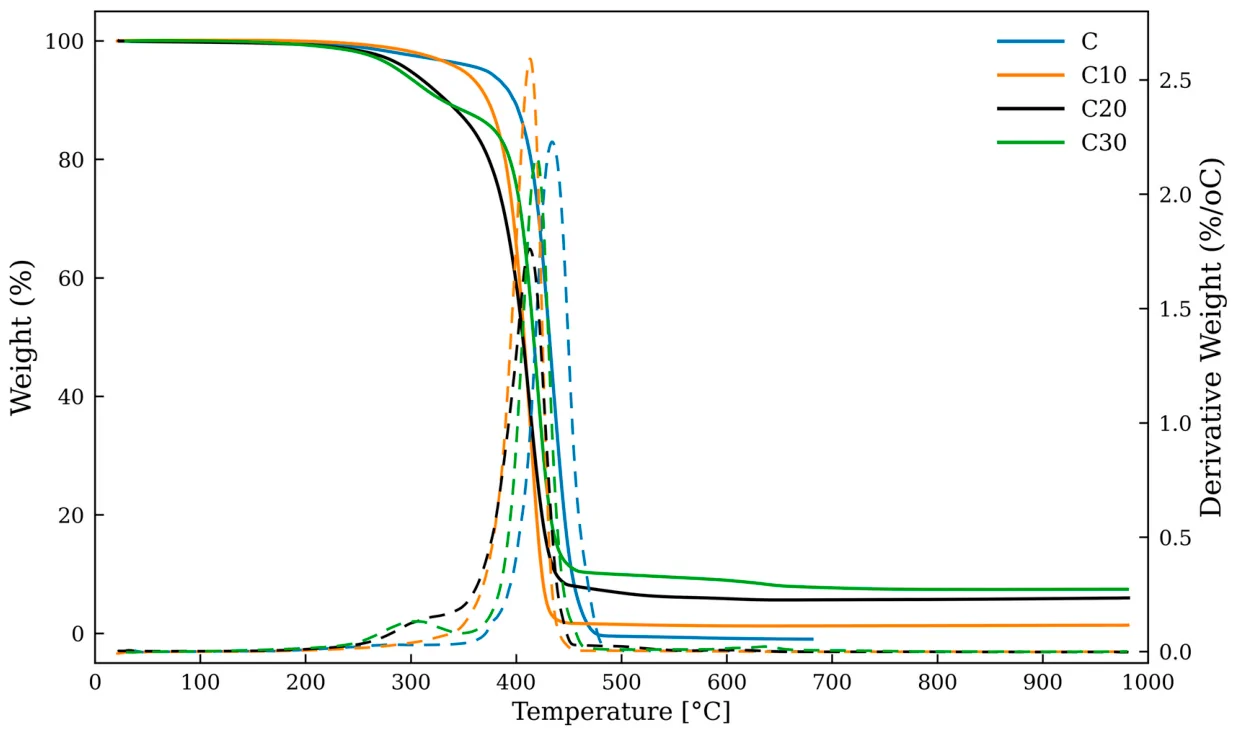
TG/DTG curves of PS-based samples (C, C10, C20, C30). Solid lines represent the TG curves, whereas dashed lines represent the DTG curves.

The clearest shift in degradation parameters toward lower temperatures was observed for PS-based systems. For neat PS (C), the Tonset, Tmax, and T50% values were 409.3, 434.1, and 431.5 °C, respectively. After the incorporation of 10% waste, these values decreased to 388.7, 413.1, and 407.4 °C, whereas for C20 they were 378.6, 412.5, and 406.2 °C, respectively (Figure 7).

For sample C30, Tonset was 393.7 °C, Tmax was 419.3 °C, and T50% was 416.4 °C. This indicates that all PS composites containing waste exhibited lower characteristic degradation temperatures than neat PS; however, the magnitude of these changes did not increase monotonically with increasing waste content. The lowest Tonset and T50% values were obtained for C20, whereas for C30 a partial increase in these parameters was observed compared with C20.

At the same time, the total mass loss decreased systematically with increasing waste content, from 100% for C to 98.60% for C10, 94.01% for C20, and 92.54% for C30. The results therefore indicate that the presence of distilled paint waste affects both the initial and the main degradation stages of PS, shifting the characteristic temperatures toward lower values relative to the neat matrix. However, it should not be concluded that this effect increases proportionally with waste content, because the changes in Tonset, Tmax, and T50% across C10–C30 are non-monotonic.

Differential scanning calorimetry (DSC) was used to evaluate the thermal transitions occurring in the investigated materials. The cooling and second-heating curves were analyzed, whereas the first heating cycle was used to erase the thermal history of the samples and was not included in the interpretation of the results. One DSC measurement was performed for each investigated system; therefore, the results are presented as individual values without statistical analysis. The glass transition temperature, Tg, was determined from the second-heating curve as the midpoint of the change in heat capacity, whereas for PP, the onset and peak temperatures of the crystallization and melting effects were additionally determined. A summary of the determined parameters is presented in Table 8.

**Figure 8:**
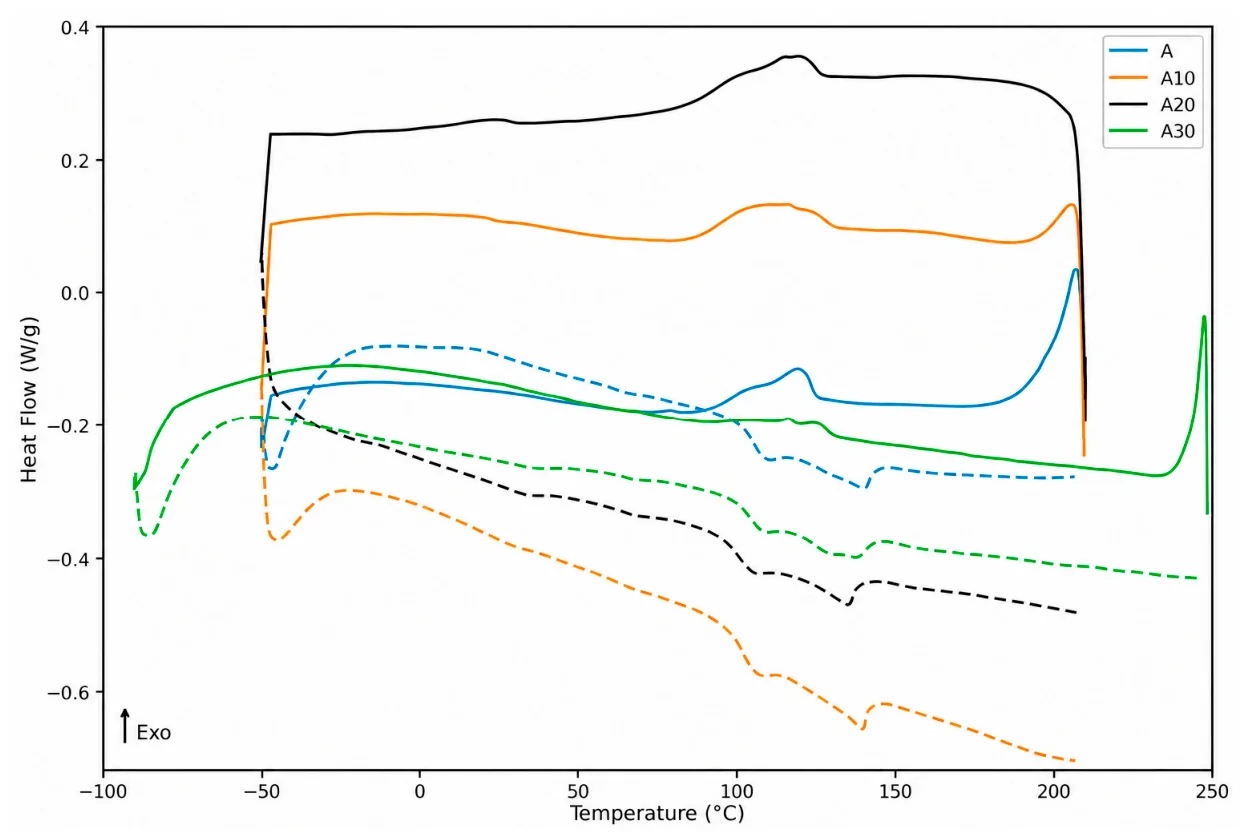
DSC curves of ABS-based samples (A, A10, A20, A30). Solid lines represent the cooling curves, whereas dashed lines represent the second heating curves.

For the ABS-based materials, the cooling curves of samples A, A10, A20, and A30 did not exhibit distinct exothermic peaks characteristic of crystallization. The observed changes appeared as broad baseline deviations, which is consistent with the amorphous nature of the matrix. During the second heating cycle, a glass transition was identified for all samples. The Tg values were 105.4 °C for A, 102.2 °C for A10, 100.8 °C for A20, and 104.0 °C for A30, respectively. The corresponding Tg, onset values were 101.7, 98.7, 97.0, and 100.0 °C, respectively (Figure 8). The lowest Tg value was obtained for A20, whereas for A30 the glass transition temperature again approached the value characteristic of unmodified ABS. The results therefore indicate that the presence of distilled paint waste modifies the segmental mobility of the amorphous ABS phase only to a limited extent. However, based on DSC analysis alone, the observed changes cannot be unequivocally attributed to a specific interaction mechanism between the waste and the polymer matrix.

**Figure 9:**
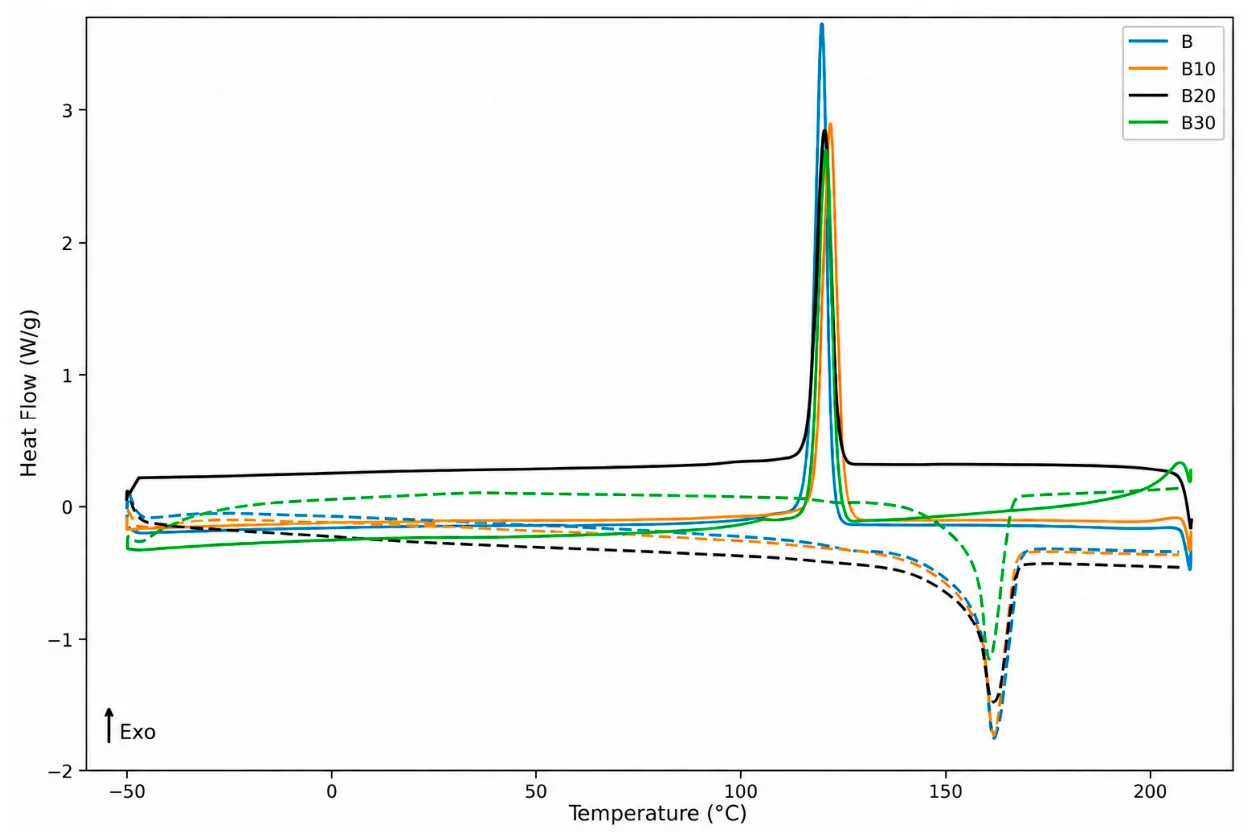
DSC curves of PP-based samples (B, B10, B20, B30). Solid lines represent the cooling curves, whereas dashed lines represent the second heating curves.

The DSC curves of the PP-based materials exhibited behavior typical of a semicrystalline polymer (Figure 9). During cooling, distinct exothermic effects associated with crystallization were observed, whereas during the second heating cycle, intense endothermic peaks corresponding to melting of the crystalline phase were present. The crystallization peak temperatures, Tc, were 119.7 °C for B, 121.7 °C for B10, 120.4 °C for B20, and 120.6 °C for B30, whereas the corresponding Tconset values were 122.0, 124.8, 123.5, and 123.6 °C, respectively. The largest shift in the crystallization peak toward a higher temperature was observed for sample B10, whereas with a further increase in waste content, the Tc value again approached that obtained for unmodified PP. Thus, no monotonic relationship was found between waste content and crystallization temperature.

During the second heating cycle, all PP samples exhibited a single dominant melting effect. The melting peak temperatures, Tm, were 161.7 °C for B, 161.7 °C for B10, 161.7 °C for B20, and 160.6 °C for B30, whereas the corresponding Tm, onset values were 158.2, 158.1, 157.0, and 156.8 °C, respectively. The variation in the melting peak temperature was therefore small. The results indicate that the addition of the waste does not eliminate the ability of the PP matrix to crystallize and melt, but results in minor changes in the course of these transitions.

**Figure 10:**
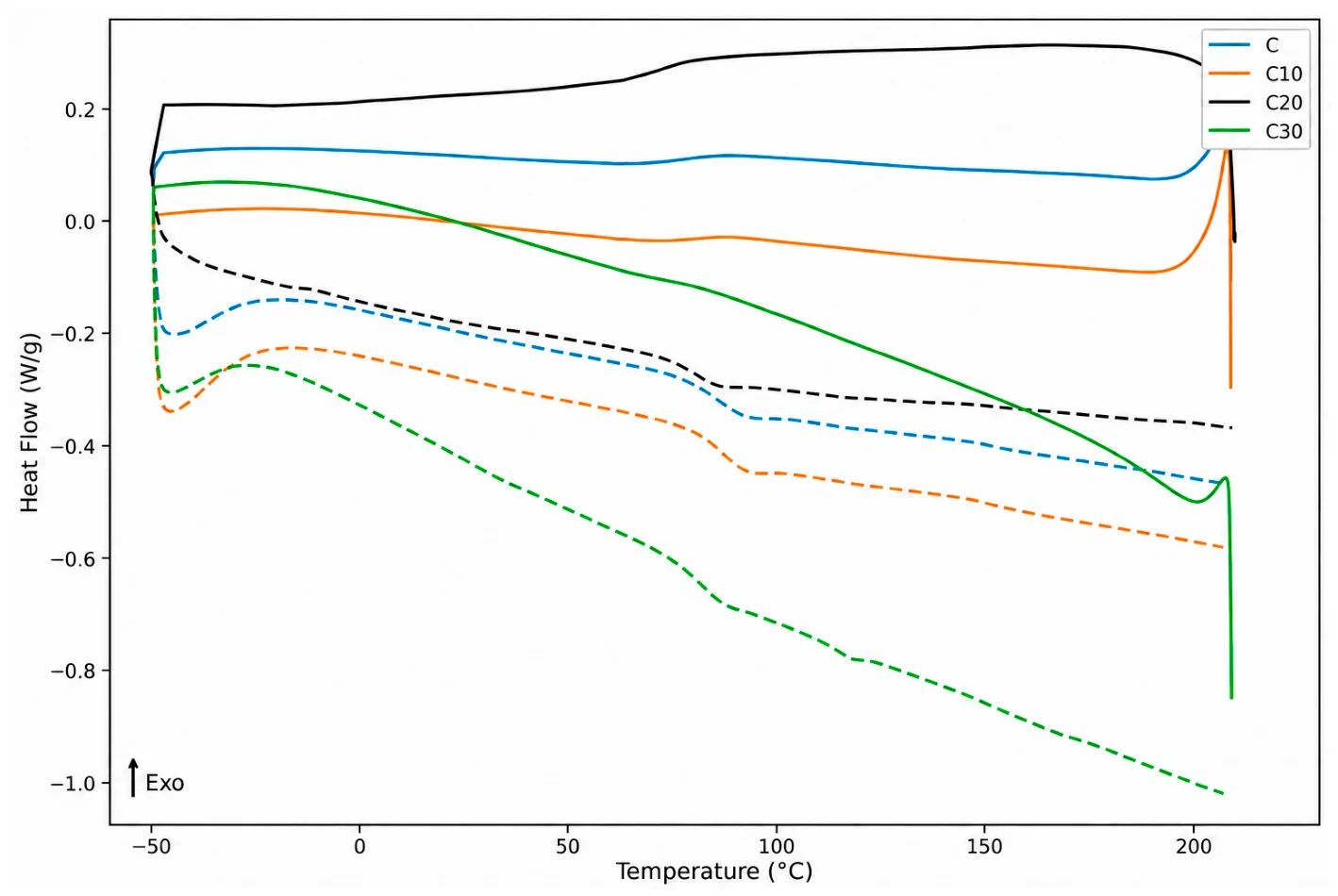
DSC curves of PS-based samples (C, C10, C20, C30). Solid lines represent the cooling curves, whereas dashed lines represent the second heating curves.

For the PS-based materials, no exothermic effects corresponding to crystallization were observed in the cooling curves of samples C, C10, C20, and C30 (Figure 10). The recorded changes were primarily manifested as a baseline shift associated with the glass transition, which is consistent with the amorphous nature of the investigated polystyrene. During the second heating cycle, a pronounced decrease in the glass transition temperature was observed after the incorporation of distilled paint waste. For the reference sample C, the Tg value of 106.4 °C was retained. For the waste-containing samples, the Tg mid-point values were 87.1 °C for C10, 80.5 °C for C20, and 81.6 °C for C30, whereas the corresponding Tgonset values were 81.1, 75.4, and 76.7 °C, respectively.

The inclusion of sample C20 makes it possible to conclude that the largest decrease in the glass transition temperature occurs between unmodified PS and the waste-containing composites. A further increase in the waste content from 20 to 30 wt.% did not result in an additional pronounced decrease in Tg, because the values obtained for C20 and C30 were similar. The observed shift inTg toward lower temperatures indicates an increase in the effective segmental mobility of the amorphous PS phase. This effect may be associated with the presence of waste constituents exerting a plasticizing effect on the matrix; however, this interpretation should be regarded as a hypothesis, because DSC analysis alone does not allow identification of the compounds responsible for the observed change. At the same time, the broader and more diffuse glass transition observed in the waste-containing samples may indicate greater heterogeneity in the local conditions governing segmental relaxation. Therefore, compared with the previous description, the unequivocal attribution of the decrease in Tg solely to a plasticization effect has been limited.

**Figure 11:**
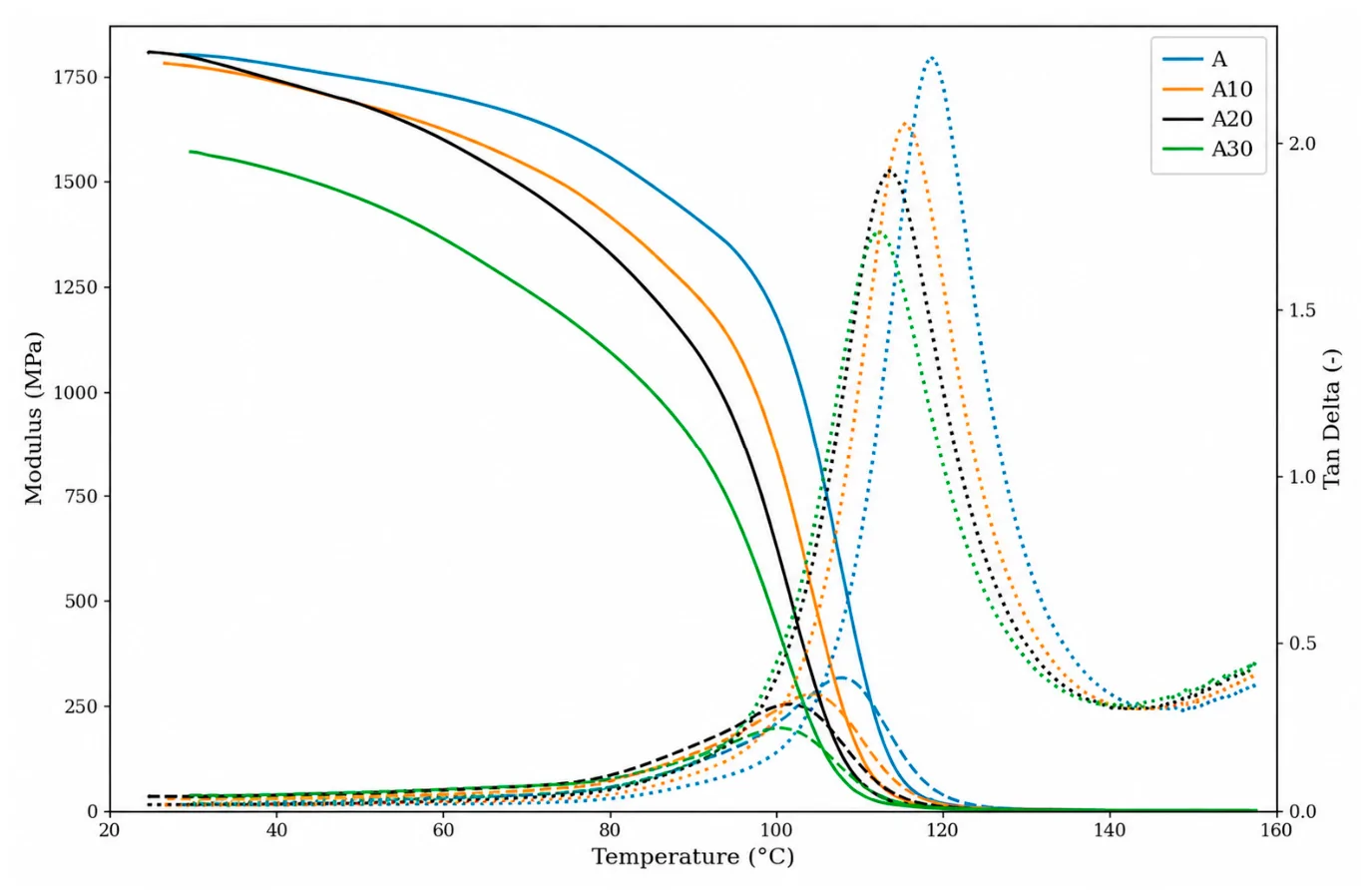
Thermomechanical curves of ABS-based samples (A, A10, A20, A30). Solid lines represent the storage modulus (E′), dash-dotted lines represent the loss modulus (E″), and dotted lines represent the loss factor (tan δ).

The results of dynamic mechanical analysis indicate that the presence of distilled paint waste affects the viscoelastic response of ABS-based composites (Figure 11). At approximately 30 °C, the storage modulus E′ values were 1802.42 MPa for sample A, 1775.48 MPa for A10, 1795.83 MPa for A20, and 1570.36 MPa for A30. Thus, the E′ values for samples A, A10, and A20 remained at a comparable level, whereas a pronounced decrease in dynamic stiffness was observed at a waste content of 30%. As the temperature increased, the differences among the investigated materials became more pronounced. At approximately 80 °C, the E′ values were 1558.02, 1417.51, 1330.20, and 1095.91 MPa for A, A10, A20, and A30, respectively, indicating a progressive reduction in the elastic contribution to the material response with increasing waste content.

Changes in the loss modulus E″ further confirm the effect of the additive on the main viscoelastic relaxation process. The maximum E″ decreased from 316.66 MPa for sample A to 277.04 MPa for A10, 253.56 MPa for A20, and 195.70 MPa for A30. At the same time, the temperature corresponding to the E″ maximum shifted toward lower values, from 107.71 °C to 104.12 °C, 101.62 °C, and 100.43 °C, respectively. A similar trend was observed for tan δ [37]. The temperature of the tan δ maximum decreased from 118.60 °C for A to 115.39 °C for A10, 113.76 °C for A20, and 112.13 °C for A30, while the maximum value simultaneously decreased from 2.254 to 2.058, 1.917, and 1.736, respectively. The results therefore indicate that increasing the waste content leads to a gradual shift in the ABS relaxation response toward lower temperatures and to a reduction in dynamic stiffness, which is particularly evident at the highest additive content.

**Figure 12:**
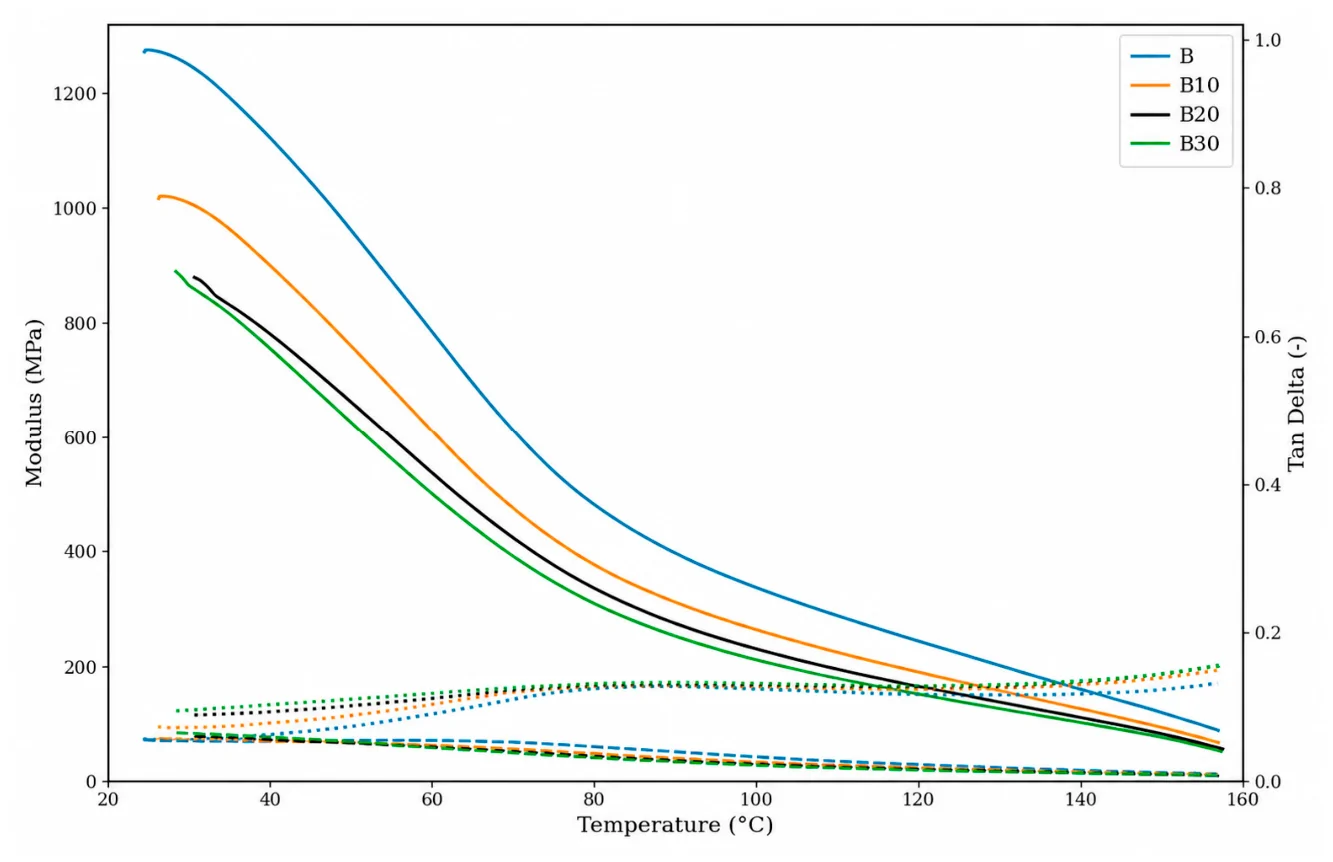
Thermomechanical curves of PP-based samples (B, B10, B20, B30). Solid lines represent the storage modulus (E′), dash-dotted lines represent the loss modulus (E″), and dotted lines represent the loss factor (tan δ).

For the PP-based composites, a systematic decrease in the storage modulus E′ was observed over the entire analyzed temperature range with increasing distilled paint waste content (Figure 12). At approximately 30 °C, E′ decreased from 1250.33 MPa for sample B to 1008.49 MPa for B10, 878.80 MPa for B20, and 866.92 MPa for B30. A similar ordering of the values was maintained at higher temperatures. At approximately 80 °C, the E′ values were 483.47, 379.40, 337.94, and 311.72 MPa, respectively, whereas at approximately 120 °C they were 244.14, 189.77, 165.47, and 151.79 MPa. The obtained results indicate a gradual reduction in the elastic contribution to the dynamic response of the material with increasing waste content. In contrast to the ABS- and PS-based systems, no common, well-defined E″ maximum that could be unequivocally attributed to the main relaxation associated with the glass transition was observed for the investigated PP series. The highest E″ values occurred near the lower limit of the analyzed temperature range or appeared as broad and weakly defined changes. Therefore, these values should not be interpreted as glass-transition temperatures. The tan δ curves exhibited broad local maxima in the range of approximately 88–90 °C, with only minor differences in their values among the individual composites. The PP results primarily indicate a reduction in dynamic stiffness with increasing waste content, whereas the nature of the changes in E″ and tan δ does not allow unambiguous conclusions to be drawn regarding either improvement or deterioration of vibration-damping capability or the service temperature of the material. The interpretation was therefore limited to the viscoelastic response recorded under the measurement conditions at a frequency of 1 Hz.

**Figure 13:**
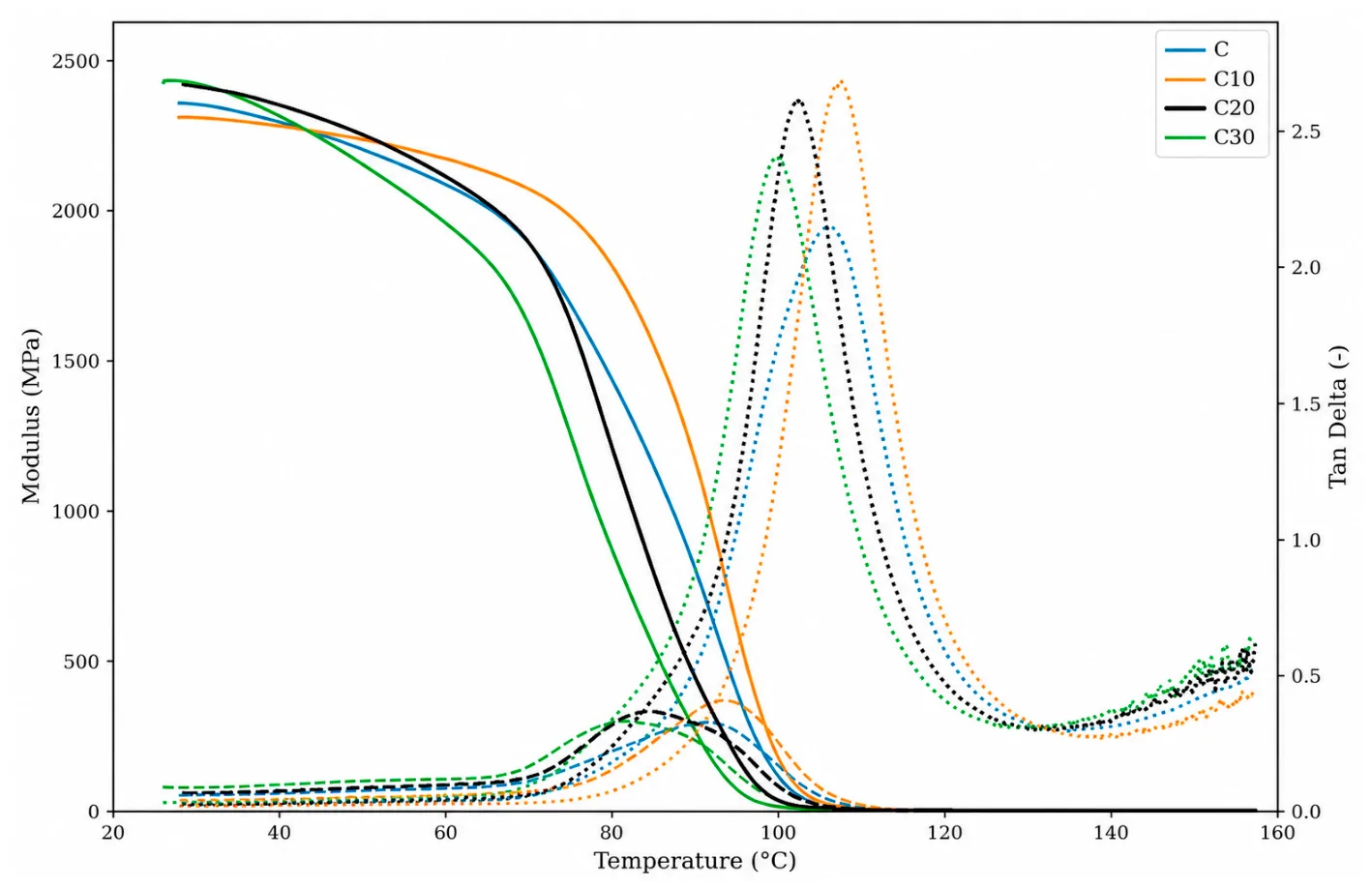
Thermomechanical curves of PS-based samples (C, C10, C20, C30). Solid lines represent the storage modulus (E′), dash-dotted lines represent the loss modulus (E″), and dotted lines represent the loss factor (tan δ).

The PS-based composites exhibited a different pattern of changes compared with ABS and PP. At approximately 30 °C, the storage modulus E′ values were 2243.25 MPa for sample C, 2310.14 MPa for C10, 2414.08 MPa for C20, and 2422.73 MPa for C30 (Figure 13). The increase in E′ with increasing waste content indicates that, within the glassy-state region, the presence of the waste phase contributes to an increase in the dynamic stiffness of the system. However, this effect was not maintained over the entire temperature range, because with increasing temperature, the decrease in E′ occurred earlier for the waste-containing composites than for neat PS.

Analysis of the loss modulus E″ indicates a clear shift in the main relaxation toward lower temperatures with increasing waste content. The temperature of the E″ maximum was approximately 96.0 °C for sample C, 93.36 °C for C10, 84.47 °C for C20, and 81.61 °C for C30. For the waste-containing samples, the maximum E″ value decreased from 370.21 MPa for C10 to 331.24 MPa for C20 and 300.14 MPa for C30. A similar trend was observed for the tan δ maximum, the temperature of which shifted from 107.38 °C for C10 to 102.49 °C for C20 and 99.75 °C for C30, while the maximum value simultaneously decreased from 2.689 to 2.612 and 2.404, respectively.

The results therefore indicate the coexistence of two effects: an increase in the dynamic stiffness of PS in the glassy state and a shift in the relaxation processes toward lower temperatures. This behavior may be associated with the contribution of the dispersed waste phase to load transfer at lower temperatures, together with an increase in the effective segmental mobility of the amorphous phase. However, based solely on the DMA measurements, this phenomenon cannot be unequivocally attributed to a specific molecular mechanism, nor can a plasticization effect be directly confirmed.

## 4. Conclusions

The present study demonstrated that the specific batch of distilled paint waste investigated can be incorporated into ABS, PP, and PS matrices at nominal waste feed contents of 10, 20, and 30 wt.% and processed by extrusion followed by injection molding. The addition of the waste affected processing behavior, mechanical performance, thermal properties, and dynamic-mechanical response, with the magnitude and direction of these changes depending strongly on the polymer matrix and nominal waste feed content.

The melt-flow behavior was clearly matrix-dependent. For ABS and PP, MFR changed non-monotonically with increasing waste feed content, whereas for PS it increased progressively from 9.52 g/10 min for neat PS to 17.28, 22.40, and 24.85 g/10 min for C10, C20, and C30, respectively. These results indicate that the waste substantially modifies melt-flow behavior; however, MFR measurements alone do not allow the mechanisms responsible for these changes to be identified. Helium pycnometry also revealed matrix-dependent changes in skeletal density.

The incorporation of distilled paint waste generally reduced the mechanical performance of the investigated materials. The strongest reduction in impact resistance was observed for the ABS-based materials, for which impact strength decreased from 17.51 kJ/m^2^ for the neat matrix to 0.23 kJ/m^2^ for A30. The maximum tensile stress of ABS decreased from 42.21 MPa for A to 26.17 MPa for A30. PP showed a more gradual decrease in both impact resistance and tensile strength. For the PS-based composites tested under the same experimental conditions, the maximum tensile stress decreased from 45 MPa for Cto 33.71 MPa for C10, 26.88 MPa for C20 and 22.26 MPa for C30. The impact strength of all PS composites nevertheless remained substantially lower than that of neat PS. The mechanical results therefore indicate that increasing nominal waste feed content generally compromises load-bearing and impact performance, although the magnitude of this effect depends on the polymer matrix.

Thermal analyses confirmed that the influence of the waste was also matrix-dependent. TGA revealed no universal monotonic relationship between waste content and thermal stability for ABS, PP, and PS. DSC showed changes in the thermal transitions of the investigated matrices, including a pronounced decrease in the glass-transition temperature of the PS-based systems. For PP, the calculated degree of crystallinity did not exhibit a simple monotonic dependence on waste content. Since the crystallinity calculations were based on the nominal waste feed fraction rather than on an experimentally verified final composite composition, these values should be regarded as approximate. Dynamic mechanical analysis additionally confirmed changes in stiffness and relaxation behavior resulting from waste incorporation.

An important limitation of the present study concerns the actual amount of waste retained in the composites after processing. TGA of the distilled paint waste showed a mass loss of 21.38% between 25 and 177 °C, i.e., within a temperature range relevant to subsequent processing. Therefore, the values of 10, 20, and 30 wt.% used in this study represent nominal waste feed contents and not experimentally verified final waste concentrations in the composites. Consequently, the actual retained fraction of waste after extrusion and injection molding remains unknown.

The conclusions of this study are limited to the specific industrial waste batch examined. The detailed chemical and mineral composition, ash content, and batch-to-batch variability of the waste were not determined. Therefore, the observed relationships should not be generalized to distilled paint waste from other sources or coating systems without additional compositional characterization and experimental verification.

## Figures and Tables

**Table 1:** Extrusion processing parameters of the investigated polymer matrices and composites.

Polymer Matrix	Sample Designation	Zone 1 [°C]	Zone 2 [°C]	Zone 3 [°C]	Zone 4 [°C]	Die [°C]
ABS	A, A10, A20, A30	165	185	195	195	195
PP	B, B10, B20, B30	165	185	195	195	195
PS	C, C10, C20, C30	165	185	195	195	195

**Table 2:** Designations of the produced composite materials.

Matrix	Nominal Distilled Paint Waste Feed Content [wt.%]			
	0	10	20	30
ABS	A	A10	A20	A30
PP	B	B10	B20	B30
PS	C	C10	C20	C30

**Table 3:** Injection molding process parameters for the polymer matrices and composites under investigation.

Polymer Matrix	Sample Designation	Zone 1 [°C]	Zone 2 [°C]	Zone 3 [°C]	Nozzle [°C]
ABS	A, A10, A20, A30	210	210	220	225
PP	B, B10, B20, B30	170	190	190	200
PS	C, C10, C20, C30	200	210	220	230

**Table 4:** Density of the investigated polymer matrices and composites [g/cm^3^].

Matrix	0% wt.	10% wt.	20% wt.	30% wt.
ABS	1.04	1.06	1.08	1.10
PP	0.90	0.93	0.96	0.98
PS	1.05	0.90	0.94	0.96

**Table 5:** Summary of MFR values [g/10 min].

Matrix	Measurement Conditions	0% wt.	10% wt.	20% wt.	30% wt.
ABS	235 °C/5 kg	18.38	23.97	24.53	20.30
PP	230 °C/2.16 kg	64.77	39.06	46.45	58.30
PS	200 °C/5 kg	9.52	17.28	22.40	24.85

**Table 6:** Selected parameters from the static tensile test.

Sample	σ_M_ [MPa]	ε_M_ [%]	σ_B_ [MPa]	ε_B_ [%]	E [MPa]
A	42.21 ± 0.73	3.46 ± 0.07	33.47 ± 2.15	7.00 ± 2.55	1557 ± 29
A10	32.27 ± 1.04	2.53 ± 0.12	31.35 ± 1.00	2.84 ± 0.35	1566 ± 36
A20	28.48 ± 2.88	2.24 ± 0.25	28.35 ± 2.80	2.28 ± 0.28	1548 ± 73
A30	26.17 ± 1.70	2.15 ± 0.18	26.16 ± 1.69	2.16 ± 0.19	1550 ± 51
B	24.81 ± 0.24	6.56 ± 0.10	19.12 ± 0.44	23.16 ± 5.15	1071 ± 18
B10	20.19 ± 0.18	7.98 ± 0.18	19.93 ± 0.19	8.49 ± 0.32	779 ± 19
B20	19.02 ± 0.41	8.78 ± 0.22	18.52 ± 0.46	10.08 ± 0.42	708 ± 48
B30	16.55 ± 0.14	8.60 ± 0.17	16.19 ± 0.09	9.90 ± 0.41	661 ± 19
C	45.0± 0.24	2.01± 0.09	39.22 ± 0.27	2.00 ± 0.18	3000 ± 95
C10	33.71 ± 1.72	1.94 ± 0.08	33.30 ± 1.45	2.17 ± 0.25	2120 ± 109
C20	26.88 ± 0.98	1.68 ± 0.13	25.65 ± 0.54	2.18 ± 0.30	2097 ± 121
C30	22.26 ± 1.11	1.49 ± 0.29	19.73 ± 0.98	2.86 ± 0.88	2053 ± 70

**Table 7:** Parameters of thermogravimetric analysis (TGA).

Sample	Tonset [°C]	Tmax [°C]	Δm [%]	T50% [°C]
Waste	25; 177; 572	89.4; 395; 721	21.4; 41.50; 9	449.4
A	398.6	423	99.97	423.9
A10	391.7	422.2	97.26	422.4
A20	391.7	425.3	94.08	425.8
A30	383.2	416.9	92.24	418.8
B	416.3	449.3	99.34	438.1
B10	432.2	460.2	97.55	452.8
B20	410.8	452.9	91.56	442.1
B30	427	461.4	92.84	452.8
C	409.3	434.1	100	431.5
C10	388.7	413.1	98.6	407.4
C20	378.6	412.5	94.01	406.2
C30	393.7	419.3	92.54	416.4

**Table 8:** Parameters of differential scanning calorimetry (DSC) analysis.

Sample	Tg, Onset [°C]	Tg, Mid [°C]	ΔCp [J/(g·K)]	Tc, Onset [°C]	Tc, Peak [°C]	|ΔHc| [J/g]	Tm, Onset [°C]	Tm, Peak [°C]	ΔHm [J/g]	Xc [%]
A	101.7	105.4	0.16	—	—	—	—	—	—	—
A10	98.7	102.2	0.23	—	—	—	—	—	—	—
A20	97.0	100.8	0.20	—	—	—	—	—	—	—
A30	100.0	104.0	0.11	—	—	—	—	—	—	—
B	—	—	—	122.0	119.7	85.3	158.6	161.6	63.0	30.4
B10	—	—	—	124.8	121.7	79.1	158.6	161.7	55.9	30.0
B20	—	—	—	123.5	120.4	69.0	157.7	161.8	48.4	29.3
B30	—	—	—	123.6	120.6	70.9	157.5	160.6	50.2	34.7
C	102.0	106.4	0.38	—	—	—	—	—	—	—
C10	81.1	87.1	0.33	—	—	—	—	—	—	—
C20	75.4	80.5	0.21	—	—	—	—	—	—	—
C30	76.7	81.6	0.25	—	—	—	—	—	—	—

Xc was calculated using the nominal waste feed fraction because the retained waste content after processing was not quantified.

## Data Availability

The original contributions presented in this study are included in the article. Further inquiries can be directed to the corresponding authors.
